# Fatty acid desaturase 2 is up-regulated by the treatment with statin through geranylgeranyl pyrophosphate-dependent Rho kinase pathway in HepG2 cells

**DOI:** 10.1038/s41598-019-46461-9

**Published:** 2019-07-10

**Authors:** Shou Tanaka, Noriko Ishihara, Sawako Suzuki, Yasuhiro Watanabe, Daiji Nagayama, Takashi Yamaguchi, Masahiro Ohira, Atsuhito Saiki, Tomoaki Tanaka, Ichiro Tatsuno

**Affiliations:** 1 Center for Diabetes, Metabolism and Endocrinology, Toho University Sakura Medical Center, Sakura, Japan; 2 Graduate School of Medicine, Toho University, Tokyo, Japan; 3 Department of Clinical Cell Biology, Graduate School of Medicine, Chiba University, Chiba, Japan; 4 Department of Molecular Diagnosis, Graduate School of Medicine, Chiba University, Chiba, Japan

**Keywords:** Experimental models of disease, Preclinical research

## Abstract

Statins have been reported to increase the plasma concentration of arachidonic acid (AA), an omega-6 long chain polyunsaturated fatty acid (LCPUFA) in several clinical studies indicating that statins affect the endogenous synthesis of LCUFAs. In the present study, we investigated the roles of the intrinsic mevalonate cascade and Rho-dependent pathway in LCPUFA synthesis, especially focusing on fatty acid desaturases (Fads) 2, using the human hepatocellular carcinoma cell line HepG2. Cell number and the activity of caspase-3 and 7 (caspase-3/7) was measured using a commercial kit. Gene expression was analyzed by quantitative real-time PCR. Protein expression was detected by Western blot analysis. Atorvastatin decreased cell viability and increased caspase-3/7 activity in a dose-dependent manner. At lower concentrations, atorvastatin stimulated both mRNA and protein expression of Fads2, and increased mRNA expression of *FADS1* and *ELVOL5*. Both mevalonate and geranylgeranyl-pyrophosphate (GGPP), but not cholesterol, fully reversed atorvastatin-induced upregulation of Fads2, and mevalonate-effected reversal was inhibited by treatment with the Rho-associated protein kinase inhibitor Y-27632. These data clearly demonstrated that in human HepG2 cells, statins affect the endogenous synthesis of LCPUFAs by regulation of not only Fads2, but also Fads1 and Elovl5, through the GGPP-dependent Rho kinase pathway.

## Introduction

Long chain polyunsaturated fatty acids (LCPUFAs) have been demonstrated to play critical roles in the regulation of many biological functions such as brain development, cognition, emotion, reproduction, inflammation, and homeostasis. Arachidonic acid (AA, 20:4 n-6), a 20-carbon 4-double-bond omega-6 LCPUFA, is an “essential fatty acid” not synthesized by the human body. AA is one of the crucial components of membrane phospholipids and the major source of eicosanoids, both of which are lipid modulators of vascular function. Eicosanoids mainly act locally by signaling via specific receptors, and modulate many functions such as vasomotor tone, hemostasis, inflammation, and cell proliferation. In the pathological setting of atherosclerosis, eicosanoids play significant roles since they contribute to endothelial dysfunction and cause inflammation, arterial smooth muscle cell hyperplasia and thrombosis^1^.

The landmark epidemiological study in Greenlandic Eskimos demonstrated the key role of fish oil (omega-3 LCPUFAs) in the prevention of atherosclerotic diseases^2^. Following this study, the health benefits of omega-3 LCPUFAs, consisting mainly of eicosapentaenoic acid (EPA, 22:5 n-3) and docosahexaenoic acid (DHA, 22:6 n-3), as part of a fatty acid-rich diet have been researched extensively. Large-scale epidemiological studies, clinical outcome trials, and meta-analyses have demonstrated significant relative cardiovascular risk reduction by intake of omega-3 LCPUFAs^3–7^. Studies have also shown that omega-3 and omega-6 LCPUFAs have opposing effects on metabolic functions in the body, and the balance between EPA or DHA and AA is likely to be important for regulating the production of mediators and subsequently vascular function in the human body. Indeed, serum EPA to AA ratio (EPA/AA) has been found to be a good biomarker for the risk of cardiovascular disease not only in the general population^8^, but also in a post-hoc analysis of the results of a clinical trial^9^.

The important roles of statins for both primary and secondary prevention of cardiovascular disease have been established^10^, and low-density-lipoprotein cholesterol (LDL-C)-lowering therapy with statins has been used as the first-line treatment. Despite significant LDL-C lowering with statins, substantial residual cardiovascular risk remains^11^, and several risk factors such as low level of high-density-lipoprotein cholesterol and high level of triglycerides have attracted attention. A particularly interesting finding is that increase in plasma AA concentration and decrease in plasma omega-3 fatty acid concentration and/or plasma omega-3/AA ratio have been observed in patients treated with statins^12–17^, indicating that statins may regulate the endogenous biosynthesis of LCPUFAs. Actually statins have been reported to increase synthesis of AA from linoleic acid (LA, 18:2 n-6) *in vivo*^18,19^. Although these effects of statins on endogenous biosynthesis of LCPUFAs may be involved in the residual risk after initiation of statin treatment, its precise molecular mechanism remains unknown.

LCPUFAs are endogenously biosynthesized from omega-6 and omega-3 LCPUFA precursors by position-specific desaturation and carbon-chain elongation reactions^20^. Endogenous synthesis of LCPUFAs and the degree of unsaturation of the biological membranes depend largely on the actions of fatty acid desaturases Fads1 (∆5-desaturase), Fads2 (∆6-/∆8-/∆4-desaturase) and putatively Fads3, as well as elongation of very long-chain fatty acids proteins (Elovls)^21^. Recent genome-wide association studies demonstrated that the genes mediating endogenous synthesis of LCPUFAs contribute to wide variability in the efficiency of LCPUFA synthesis, and that synthesis is likely controlled at the levels of Fads2 and Fads1 as well as by the elongases depending on genotype and metabolic state^21^.

Since Fads2 is known to play an important role in endogenous biosynthesis of AA, EPA and DHA^21^, we studied the effect of atorvastatin on gene and protein expression of this gene, and its regulatory mechanism via the mevalonate cascade. We used human hepatocellular carcinoma cell line HepG2 in this study, because the liver is the major organ for endogenous biosynthesis of LCPUFAs^22^, and HepG2 cells have been used extensively in studies of human liver lipid and lipoprotein metabolisms^23^.

## Results

### Effect of atorvastatin on HepG2 cell viability

**Figure 1:**
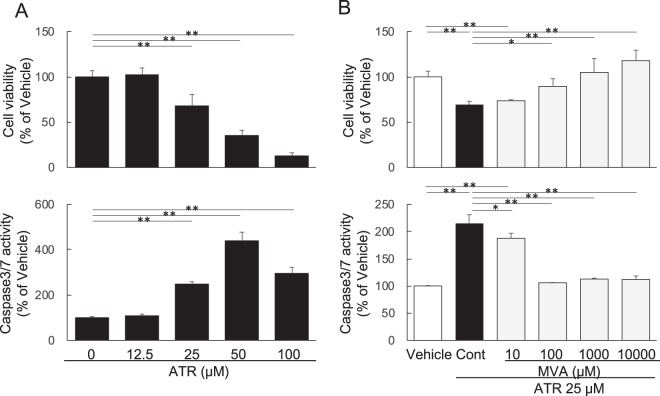
Effects of atorvastatin on cell viability and caspase3/7 activity in HepG2 cells. (**A**) Dose-dependent effect of atorvastatin. (**B**) Effect of mevalonate under treatment with atorvastatin (30 μM). ATR: atorvastatin, MVA: mevalonolactone. Data are presented as mean ± SD of 5 experiments. *p < 0.05, **p < 0.01; one-way ANOVA followed by Bonferroni multiple comparison test.

At 48 h, ATR decreased cell viability significantly to 68.0 ± 5.7% at 25 μM, 35.3 ± 2.6% at 50 μM and 12.8 ± 1.5% at 100 μM, apparently in a dose-dependent manner (Fig. 1A). On the contrary, caspase 3/7 activity was significantly stimulated by atorvastatin at 25 μM, and peaked at 50 μM (Fig. 1A).

Both ATR-induced decease in cell viability and increase in caspase 3/7 activity were reversed by the addition of MVA in a dose-dependent manner as shown in Fig. 1B, although MVA did not affect cell viability and caspase 3/7 activity in the absence of ATR (data not shown).

### Effect of atorvastatin and mevalonate on FADS1, FADS2, and ELOVL5 expression in HepG2 cells

**Figure 2:**
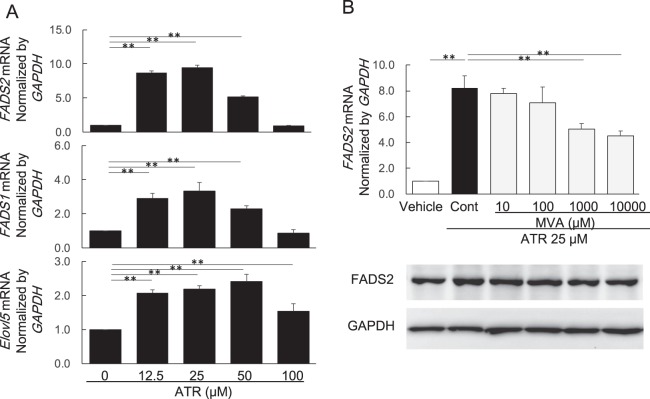
Effects of atorvastatin and mevalonate on *FADS1*, *FADS2*, and *ELOVL5* mRNA and protein expression in HepG2 cells. (**A**) Dose-dependent effect of atorvastatin on *FADS2*, *FADS1* and *ELOVL5* mRNA expression. (**B**) Effect of mevalonate on *FADS2* mRNA and FADS2 protein expression in HepG2 cells treated with ATR (25 μM). The grouping of gel/blots cropped from different gels. ATR: atorvastatin, MVA: mevalonolactone, Data are presented as mean ± SD of 6 experiments. *p < 0.05, **p < 0.01; one-way ANOVA followed by Bonferroni multiple comparison test.

We examined the effects of ATR on mRNA expression of *FADS1*, *FADS2* and *ELOVL5* in HepG2 cells (Fig. 2A). *FADS2* mRNA expression increased markedly by the addition of ATR at lower concentrations (871 ± 11.3% at 12.5 μM and 945 ± 15.0% at 25 μM, relative to control), and was followed by a decline at higher concentrations of ATR (517 ± 6.3% at 50 μM, and 91.1 ± 2.8% at 100 μM, relative to control). The decline in *FADS2* mRNA expression at higher concentrations of ATR appeared to be associated with decreased cell viability (Fig. 1A). Similarly, ATR-induced increases in *FADS1* mRNA expression (292 ± 29.3% at 12.5 μM, 334 ± 50.9% at 25 μM, 229 ± 18.6% at 50 μM, and 86.8 ± 20.7% at 100 μM) and *ELOVL5* mRNA expression (208 ± 9.2% at 12.5 μM, 219 ± 10.0% at 25 μM, 241 ± 21.2% at 50 μM, and 154 ± 22.3% at 100 μM) were observed (Fig. 2A). These decreased expression of *FADS1*, *FADS2*, *and ELOVL5* at higher concentrations of ATR might be linked with its cell toxicity.

The effects of MVA on ATR-stimulated expression of *FADS2* mRNA and protein expression were examined. While ATR at 25 μM stimulated *FADS2* mRNA and protein expression, MVA at higher concentrations (1000 and 10000 μM) reversed the upregulation of both mRNA and protein expression in a dose-dependent manner (Fig. 2B). The fulll length gels of protein expression in Western blot analysis were shown in supplementary information.

### Effect of isoprenoids and Rho kinase inhibitor Y-27632 in HepG2 cells treated with ATR

**Figure 3:**
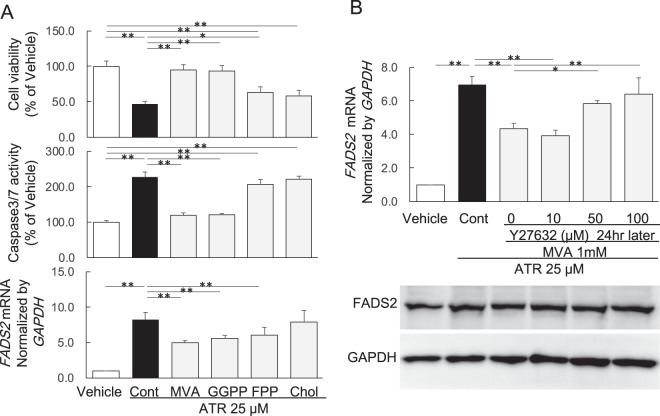
Effects of isoprenoids and Rho kinase inhibitor Y-27632 in HepG2 cells treated with ATR. (**A**) Effect of isoprenoids on cell viability, caspase3/7 activity, and *FADS2* mRNA expression in HepG2 cells treated with ATR (25 μM). Data are expressed as mean ± SD of 6 experiments. ATR: atorvastatin, MVA: mevalonolactone, GGPP: geranylgeranyl pyrophosphate, FPP: farnesyl pyrophosphate, Chol: cholesterol. (**B**) Effect of the Rho kinase inhibitor Y-27632 on *FADS2* mRNA and FADS2 protein expression in HepG2 cells treated with ATR (25 μM) and MVA (1 mM). The grouping of gel/blots cropped from different gels. Data are expressed as mean ± SD of 3 experiments. *p < 0.05, **p < 0.01; one-way ANOVA followed by Bonferroni multiple comparison test.

Since ATR affected cell viability, caspase3/7 activity and *FADS2* mRNA expression, we examined the roles of metabolites in the mevalonate cascade. Although cholesterol did not affect the ATR-induced decrease in cell viability or increase in caspase3/7 activity or *FADS2* mRNA expression, 1 mM of MVA and 10 μM of GGPP both reversed all the ATR-induced changes in cell viability, caspase3/7 activity and *FADS2* mRNA expression, and 10 μM of FPP reversed some of these changes (Fig. 3A).

The above results showed that replenishment of MVA and GGPP in culture medium reversed the ATR-induced changes, suggesting that GGPP may be depleted by ATR. Since GGPP is responsible for posttranslational activation of the small GTPase Rho A, it is possible that ATR-induced depletion of GGPP leads to inhibition of Rho A and its effector Rho-associated protein kinase (ROCK), one of the major downstream targets of Rho A. Therefore, we examined the effect of Y-27632, a Rho kinase inhibitor. We found that Y-27632 dose-dependently inhibited the MVA-effected reversal of ATR-induced upregulation of *FADS2* mRNA and protein expression in HepG2 cells (Fig. 3B). The fulll length gels of protein expression in Western blot analysis were shown in supplementary information.

### Effect of LCPUFAs in HepG2 cells treated by ATR

**Figure 4:**
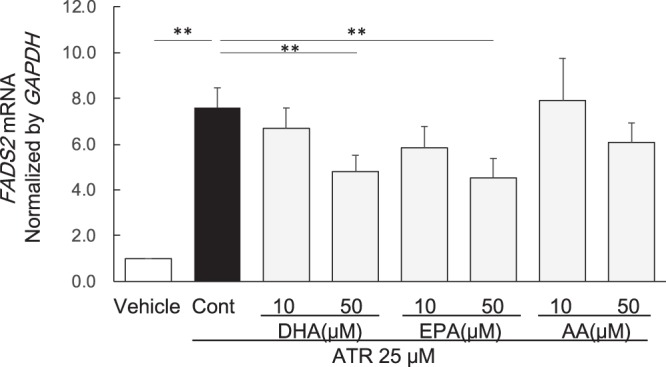
Effects of DHA, EPA, and AA on *FADS2* mRNA expression in HepG2 cells treated with ATR. ATR: atorvastatin, DHA: docosahexaenoic acid, EPA: eicosapentaenoic acid, AA: arachidonic acid. Data are expressed as mean ± SD of 6 experiments. *p < 0.05, **p < 0.01; one-way ANOVA followed by Bonferroni multiple comparison test.

Since upregulated Fads1, Fads2, and Elovl5 expression may accelerate the metabolism of both omega-3 and omega-6 LCPUFAs, we studied the effect of LCPUFAs such as AA (omega-6), EPA and DHA (both omega-3) on the ATR-induced upregulation of *FADS2* mRNA expression in HepG2 cells. EPA and DHA both significantly suppressed *FADS2* mRNA expression, but AA did not (Fig. 4).

## Materials and Methods

### HepG2 cell culture

Human hepatocellular carcinoma cell line HepG2 was obtained from the JCRB Cell Bank (Osaka, Japan), and cultured in basal medium consisting of Dulbecco’s modified Eagle’s medium (DMEM; Gibco, Thermo Fisher Scientific, Inc., Waltham, MA, USA) supplemented with 10% fetal bovine serum (FBS; Hyclone; GE Healthcare Life Sciences, Logan, UT, USA) and 100 IU/mL penicillin-streptomycin (Gibco, Thermo Fisher Scientific, Inc.) at 37 °C in 5% CO_2_ atmosphere. For the experiments, the basal medium was changed to the experimental medium (DMEM containing 0.5% FBS). Cells were exposed to atorvastatin (ATR; Wako Pure Chemical Industries, Ltd., Osaka, Japan) for 48 h in combination with various agents: mevalonolactone (MVA), geranylgeranyl-pyrophosphate (GGPP), farnesyl pyrophosphate (FPP), cholesterol, DHA, EPA, and AA (all from Sigma-Aldrich; Merck KGaA). To examine the involvement of Rho kinase signaling, the cells were also incubated with Y27632 (Wako Pure Chemical Industries, Ltd.), the selective Rho-associated protein kinase inhibitor, for 24 h after exposure to ATR and MVA.

### Cell number and assay of caspase activity

Cell number was assessed at 48 h following the addition of various reagents, by measuring mitochondrial activity that reduces 2-(2-methoxy-4-nitrophenyl)-3-(4-nitrophenyl)-5-(2,4-disulfophenyl)-2H-tetrazolium monosodium salt (WST-8) to formazan, using the Cell Counting Kit-8 assay (Dojindo Molecular Technologies, Inc., Kumamoto, Japan), according to manufacturer’s protocol.

The activity of caspase-3 and 7 (caspase-3/7) was assessed at 48 h following the addition of various reagents, using the Caspase-Glo 3/7 assay kit (Promega, Corp., Madison, WI) according to manufacturer’s protocol. The assay uses a DEVD-linked luminogenic substrate that is cleaved upon exposure to active caspase 3/7. Cleavage of the substrate results in a luminescent signal generated by activated luciferase.

### Quantitative real-time PCR

Total cellular RNA was extracted from HepG2 cells using the RNeasy kit (Qiagen S.A.S., Courtaboeuf, France). The RNA concentration of each sample was determined spectrophotometrically by measuring absorbance at 260 nm. Reverse transcription was performed using the PrimeScript^®^ RT reagent kit (Takara Bio, Inc., Otsu, Japan). The temperature protocol used was as follows: reverse transcription at 37 °C for 15 min, inactivation of reverse transcriptase by heat treatment at 85 °C for 5 s, and storage at 4 °C. Quantification of mRNA expression was performed using SYBR® Premix Ex Taq™ II (Takara Bio, Inc.) on a Mx3005P qPCR system (Agilent Technologies Japan, Ltd., Tokyo, Japan). Thermocycling conditions were as follows: enzyme activation at 95 °C for 30 s, initial denaturation at 95 °C for 5 s, followed by 40 cycles of annealing/extension at 60 °C for 30 s. This was followed by a melt curve analysis according to the manufacturer’s protocol to ensure specific amplification. The following primers were used: human *FADS1* sense, 5′-CAG CTT TGA GCC CAC CAA GAA-3′ and antisense, 5′-AGC AAG ATG TGC AGC AGG TAC AG-3′; human *FADS2* sense, 5′-TCA TGA CCA TGA TCG TCC ATA AGA A-3′ and antisense, 5′-GCT CCC AGG ATG CCG TAG AA-3′; human *ELVOL5* sense, 5′-TGA GGC AGT GGT CAA ACA GGT A-3′ and antisense, 5′-AGA TAT GTC ATG AGT GGT TCC AAGA-3′; human *GAPDH* sense, 5′-GCA CCG TCA AGG CTG AGA AC-3′ and antisense, 5′-TGG TGA AGA CGC CAG TGGA-3′; and *β-actin* sense, 5′-TGGCACCCAGCACAATGAA-3′ and antisense 5′-CTAAGTCATAGTCCGCCTAGAAGCA-3′. In the present study, GAPDH expression was used as an internal standard as reported by others^24^, since we compared the gene expression of *GAPDH* and *β-actin* as internal standards, and both expression was closely correlated in the preliminary experiment. Relative mRNA expression was calculated using the comparative Cq method and was normalized to *GAPDH* expression^25^.

### Western blot analysis

Whole-cell extracts were prepared in 1× RIPA buffer (Cell Signaling Technology, Inc., Danvers, MA, USA) supplemented with 1 mM PMSF (Cell Signaling Technology, Inc.). Lysates were sonicated briefly and cleared by full-speed centrifugation for 10 min at 4 °C. Protein quantification was performed using the Pierce BCA Protein Assay Kit (Thermo Fisher Scientific, Rockford, IL, USA). Equal amounts of protein (20 to 60 μg) were denatured and resolved by SDS-PAGE on a 10% acrylamide gel, and transferred to PVDF membranes. The membranes were incubated with the following primary antibodies: rabbit antiserum against human FADS2 (1:500; Abcam) and mouse antiserum against human GAPDH (1:5000; Wako Pure Chemical Industries Ltd., Osaka, Japan) as negative control, followed by the following HRP-conjugated secondary antibodies: donkey antiserum against rabbit IgG (1:2000; (GE Healthcare BioSciences, Little Chalfont, UK) and sheep antiserum against mouse IgG (1:2000; GE Healthcare). Bound antibodies were visualized using ECL Prime Western Blotting Detection Reagent (GE Healthcare) and imaged with the ChemiDoc XRS + imaging system (BioRad, Munich, Germany). The fulll length gels of protein expression in Western blot analysis were shown in supplementary information.

### Statistical analysis

All data are expressed as mean ± standard deviation (SD). SPSS software (version 11.5, Chicago, IL, USA) was used for statistical processing. Treatment effects were evaluated using one-way ANOVA followed by Bonferroni multiple comparison test, and *p* values less than 0.05 were considered significant.

## Discussion

The main objective of the current study was to verify whether the gene and protein expression of Fads2 is affected by treatment with ATR, a HMG-CoA reductase-specific inhibitor^26^, and the molecular mechanism involved. This study using HepG2 cells demonstrated that ATR at lower concentrations (12.5 and 25 μM) markedly upregulated the gene and protein expression of Fads2, although the upregulation was attenuated at higher concentrations of ATR (50 and 100 μM), coinciding with markedly decreased cell viability at those concentrations, consistent with previous report^27^. Atorvastatin at lower concentration also increased mRNA expression of *FADS1* and *ELVOL5*. Moreover, addition of mevalonate or its intermediate metabolite GGPP to cell culture reversed the ATR-induced upregulation of Fads2 and decrease of cell viability, suggesting that the ATR-induced changes may be caused by depletion of mevalonate and GGPP in the cells. Since GGPP is responsible for the posttranslational activation of Rho A, ATR-induced depletion of GGPP probably leads to inhibition of Rho A and its effector ROCK. Indeed, we observed that treatment with a ROCK inhibitor (Y-27632) reversed the MVA-effected suppression of *FADS2* gene expression in HepG2 cells treated with ATR. In addition, ATR-induced upregulation of *FADS2* gene was suppressed by treatment with EPA and DHA, but not AA.

The effect of statins on endogenous synthesis of LCPUFAs was first reported by Hrboticky *et al*.^18^. Using human monocytic Mono Mac 6 (MM6) cells and human hepatoma Hep G2 cells, they found that lovastatin increased AA levels to stimulate thromboxane synthesis, indicating that statin regulates the endogenous biosynthesis of LCPUFAs. Similar change in endogenous synthesis of LCPUFAs induced by simvastatin was also reported^19^. The *in vivo* effect of statin on endogenous synthesis of PUFAs was demonstrated in patients treated with simvastatin for 2 months, and simvastatin treatment was associated with an increase in AA content in erythrocyte membrane^28^. In Japanese patients with hyperlipidemia, plasma AA concentration increased significantly and EPA to AA ratio decreased significantly following statin treatment^29^. Similar results have been confirmed by other investigators^12–16,30^. Although these data strongly indicate upregulation of endogenous biosynthesis of LCPUFAs by statins, the molecular mechanism remains unknown.

Since endogenous synthesis of LCPUFAs depends largely on the actions of Fads and Elovls proteins^21^, the regulation of gene and protein expression of these enzymes may be critical for endogenous synthesis of LCPUFAs. For example, a meta-analysis of 51 human studies on the LCPUFA contents in plasma, erythrocyte or adipose tissue has shown that the proportion of DHA (22:6 n-3) is 37% lower in men than in women^31^, indicating the important roles of sex hormone in endogenous synthesis of LCPUFAs. An *in vitro* study demonstrated that progesterone upregulated omega-3 LCPUFA biosynthesis by increasing the mRNA expression of genes involved in this pathway in human liver cells^32^. Therefore, it is conceivable that statins may affect the expression and functions of Fads and Elovls. In the present study, we clearly demonstrated upregulation of gene and protein expression of *FADS2* by atorvastatin in HepG2 cells. Previous study has also shown that simvastatin increases *FADS1* and *FADS2* gene expression in human lymphoblasts^33^.

Molecular mechanism of statin-induced upregulation of Fads2 in HepG2 cells remains unclear. Statin-induced suppression of cell proliferation and viability, and statin-induced apoptosis have been reported to be dependent on mevalonic acid and GGPP in various cell types^26,34,35^ including HepG2 cells^27^. In the present study, we also demonstrated that co-incubation of HepG2 cells with MVA and its metabolite GGPP, but not cholesterol, fully reversed atorvastatin-induced decrease in cell viability and upregulation of *FADS2* gene expression. In addition, the ROCK inhibitor Y-27632 abolished the MVA-effected reversal of atorvastatin-induced upregulation of *FADS2* gene in HepG2 cells, which is consistent with our recent report that atorvastatin increases *FADS1*, *FADS2* and *ELVOL5* gene expression via the GGPP-dependent Rho kinase pathway in 3T3-L1 cells^36^. We used 3T3-L1 adipocytes in our previous study^34^ because these cells are known to have a functional Fads pathway. However, endogenous biosynthesis of LCPUFAs is known to vary in various cell systems^18,19^. We therefore examined HepG2 cells in this study and found that atorvastatin also increased not only *FADS2*, but also *FADS1* and *ELOVL5* gene expression in a GGPP-dependent manner in these cells (data not shown).

**Figure 5:**
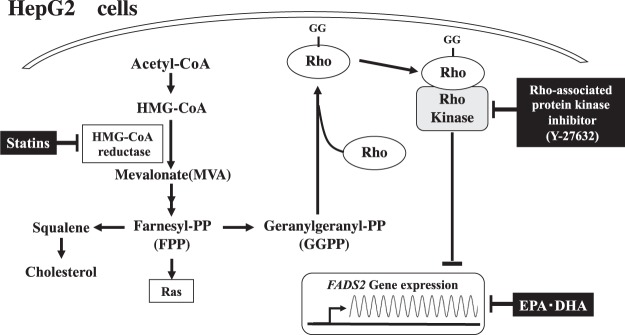
Proposed mechanism of statin-induced upregulation of *FADS2* gene expression via geranylgeranyl pyrophosphate-dependent Rho kinase pathway.

Our present study clearly demonstrates that statin upregulates protein and gene expression of *FADS2* probably through the GGPP-dependent ROCK pathway in HepG2 cells (Fig. 5), in a similar manner to a recent report of fibulin-2 expression in human coronary artery smooth muscle cells, in which simvastatin increased mRNA level of the ECM protein fibulin-2 through Rho A and Rho kinase-mediated pathway^37^. Rho A/ROCK signaling pathways play central roles in regulating cell adhesion, migration, motility, contraction, apoptosis and proliferation^38,39^. Rho A/ROCK signaling pathway is capable of controlling gene and protein expressions^40,41^, although the mechanism of how RhoA/ROCK affects protein and gene expression of *FADS2* is not known from the present study.

It is well known that the activity of Fads is primarily regulated at the transcriptional level by transcription factors such as SREBP 1c^42^, PPAR α^43,44^, and Elk^45^. From this point of view, there were the quite interesting studies, in those the statin-induced inhibition of the Rho-signaling pathway was reported to activate PPAR α activity^46^, and the treatment of statin increased the *PPARα* mRNA expression in HepG2 cells^47^. Although the mRNA expression of neither *SREBP 1c*, *PPARα*, nor *Elk* was not affected by the treatment of ATR in our study (data not shown), the involvement of PPARα remained to be possible since we did study the PPAR α activity in this study.

Another interesting finding in the present study is that EPA and DHA, both omega-3 LCPUFA, significantly suppressed the atorvastatin-induced *FADS2* gene upregulation, whereas AA, an omega-6 LCPUFA, showed a tendency of suppression, but was not significant. It is comprehensible that the downstream metabolites of Fads2, such as EPA and DHA, suppress the atorvastatin-induced upregulation of Fads2 in a negative feedback manner, but it is unclear why there is a difference in effect among the downstream metabolites of LCPUFAs (EPA and DHA versus AA).

Recently, the dietary effects of omega-3 and omega-6 LCPUFAs [studied using food with different linoleate/ α-linolenate (omega-6/omega-3) ratios] on gene expression of *FADS1*, *FADS2*, *ELVOL2* and *ELVOL5*, as well as LCPUFA composition of liver membrane phospholipids (PLs) were studied in wild type mice (WT) and heterozygous Fads2-null-mice (HET)^48^. In that study, three types of food with omega-6/omega-3 ratio of 1;1 (control), 7:1 (moderate), and 44:1 (high) were used. A diet with high omega-6/omega-3 ratio dramatically affected liver PLs; increasing AA and decreasing EPA and DHA in a dose-dependent manner. Moreover, the decrease of EPA was markedly greater than the decrease of DHA in both WT and HET mice. Specifically, AA concentration in liver PLs of mice fed a diet high in omega-6/omega-3 ratio (44:1) was three times higher compared to control diet, and EPA and DHA in liver PLs were reduced to 2.5% and 40.7%, respectively, compared to control diet in WT mice. On the contrary, a diet high in omega-6/omega-3 ratio significantly increased the gene expression of *FADS1*, *FADS2*, *ELVOL2* and *ELVOL5* in a dose-dependent manner in the liver of both WT and HET mice. These data indicate that deficiency of omega-3 in the diet causes a reduction of EPA and DHA in liver PLs to increase gene expression of *FADS1*, *FADS2*, *ELVOL2* and *ELVOL5* in the liver. The greater reduction of EPA than DHA may indicate that DHA has physiologically important roles to regulate homeostasis among the omega-3 metabolites. These findings suggest that omega-3 metabolites such as EPA and DHA, may be strongly involved in upregulation of the transcription of Fads and Elovl. Their findings may account for our result that EPA and DHA, but not AA, suppressed the atorvastatin-induced upregulation of these genes.

From the clinical point of view, the observations of increase in plasma AA concentration and decreases in plasma omega-3 LCPUFA concentration and/or omega-3/AA ratio in patients treated with statins are particularly interesting^12–16,29,30^. Although statins upregulate *FADS1*, *FADS2* and *ELVOL5* gene expression to increase endogenous synthesis of LCPUFAs (Fig. 5), it remains unclear from the present study why statin treatment predominantly increases omega-6 (AA), but reduces omega-3 (EPA and DHA) LCPUFAs in patients. There are several possibilities. The substrates for synthesis of omega-6 PUFAs, such as linoleic acid (LA, 18:2n-6), are present in much larger quantities than those for synthesis of omega-3 LCPUFAs, such as α-linolenic acid (ALA, 18:3n-3), in both *in vivo* and *in vitro* systems. As a result, conversion to AA may be dominant. This possibility is supported by a report that α-linoleic acid supplementation reduces lovastatin-induced elevation in AA and increases in cellular lipoprotein EPA and DHA levels in Hep G2 cells^49^. The other possibility is that syntheses of omega-6 and omega-3 PUFAs differ dependent on cell type. In one report, LA, ALA and stearic acid were metabolized differently in THP-1 and HepG2 cells, and simvastatin increased their conversion to a less extent in HepG2 than in THP-1, although the precise mechanism was unknown^19^.

The AA-dominant endogenous synthesis of LCPUFAs resulting in decreased plasma omega-3 concentration and/or omega-3/AA ratio during statin treatment may be clinically very important, because serum EPA/AA ratio has been reported to be a good biomarker of the risk of cardiovascular disease not only in the general population^8^, but also in a post-hoc analysis of the results of a clinical trial^9^. Therefore, it seems reasonable to recommend omega-3 LCPUFAs supplementation for patients on statin treatment, in order to maintain the plasma omega-3 concentration and omega-3/AA ratio, and to suppress the atorvastatin-induced upregulation of Fads and Elovl as demonstrated in the present study. Actually, a clinical study demonstrated the effectiveness of EPA in reducing cardiovascular events in Japanese patients treated with statin^4^.

The present study has some limitations. We used HepG2 cells as an *in vitro* model to investigate the endogenous synthesis of LCPUFAs, since it is well known that LCPUFAs in plasma are largely produced by the liver, and hepatic regulation of endogenous synthesis of LCPUFA may be critical. Since HepG2 cell is a cell line derived from hepatoblastoma, and it might be possible that the cancer cell line has the alterations in the metabolic pathways, our data should be evaluated with the consideration from these points. Therefore, it might be good to use the immortalized hepatocyte cell line in future, and it is also necessary to demonstrate statin-induced upregulation of *FADS1*, *FADS2*, and *ELVOL5* gene expression via the GGPP-dependent Rho kinase pathway in an *in vivo* model. In addition, another possibility that statin upregulates hepatic lipoprotein clearance to increase delivery of the fatty acids has been reported^18^. Although we focused on the lower concentrations of ATR due to the reduction of cell viability at its higher concentrations, the study of *FADS2* expression and its molecular mechanism at higher concentrations of ATR should have been remained.

In conclusion, statin may affect the endogenous synthesis of LCPUFAs by regulating *FADS1*, *FADS2* and *ELVOL5* gene expression via the GGPP-dependent Rho kinase pathway in HepG2 cells.

## Supplementary information

Supplymentary information

## Data Availability

The data that support the findings of this study are available from the corresponding author upon reasonable request.
