# Supplementary material for: Fatty acid desaturase 2 is up-regulated by the treatment with statin through geranylgeranyl pyrophosphate-dependent Rho kinase pathway in HepG2 cells
Source: Sci Rep. 2019 Jul 10;9:10009. doi: 10.1038/s41598-019-46461-9 (PMC6620338; doi:10.1038/s41598-019-46461-9)

Supplementary information

Fatty acid desaturase 2 is up-regulated by the treatment with statin through geranylgeranyl pyrophosphate-dependent Rho kinase pathway in HepG2 cells

Shou Tanaka[a], Noriko Ishihara[a], Sawako Suzuki[b], Yasuhiro Watanabe[a], Daiji Nagayama[a], Takashi Yamaguchi[a], Masahiro Ohira[a], Atsuhito Saiki[a], Tomoaki Tanaka[c], Ichiro Tatsuno[a]*

[a]Center for Diabetes, Metabolism and Endocrinology, Toho University Sakura Medical Center, Sakura, and Graduate School of Medicine, Toho University, Tokyo;

[b]Department of Clinical Cell Biology, Graduate School of Medicine, Chiba University, Chiba;

[c]Department of Molecular Diagnosis, Graduate School of Medicine, Chiba University, Chiba;

Supplementary Information

We show the full length gel of FADS2 and GAPDH in figure 2 and 3.

Fig2 FADS2

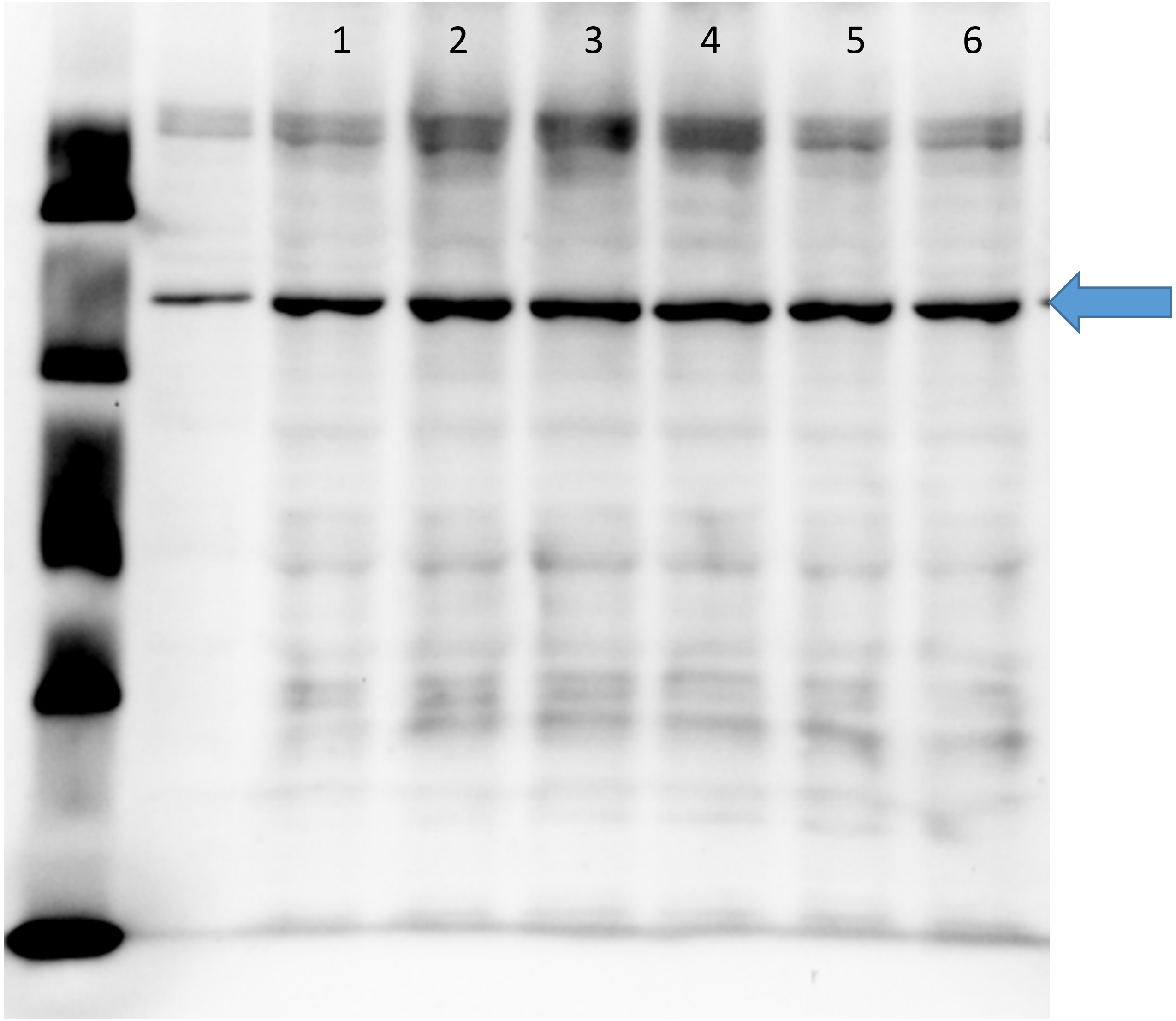

Fig2 GAPDH

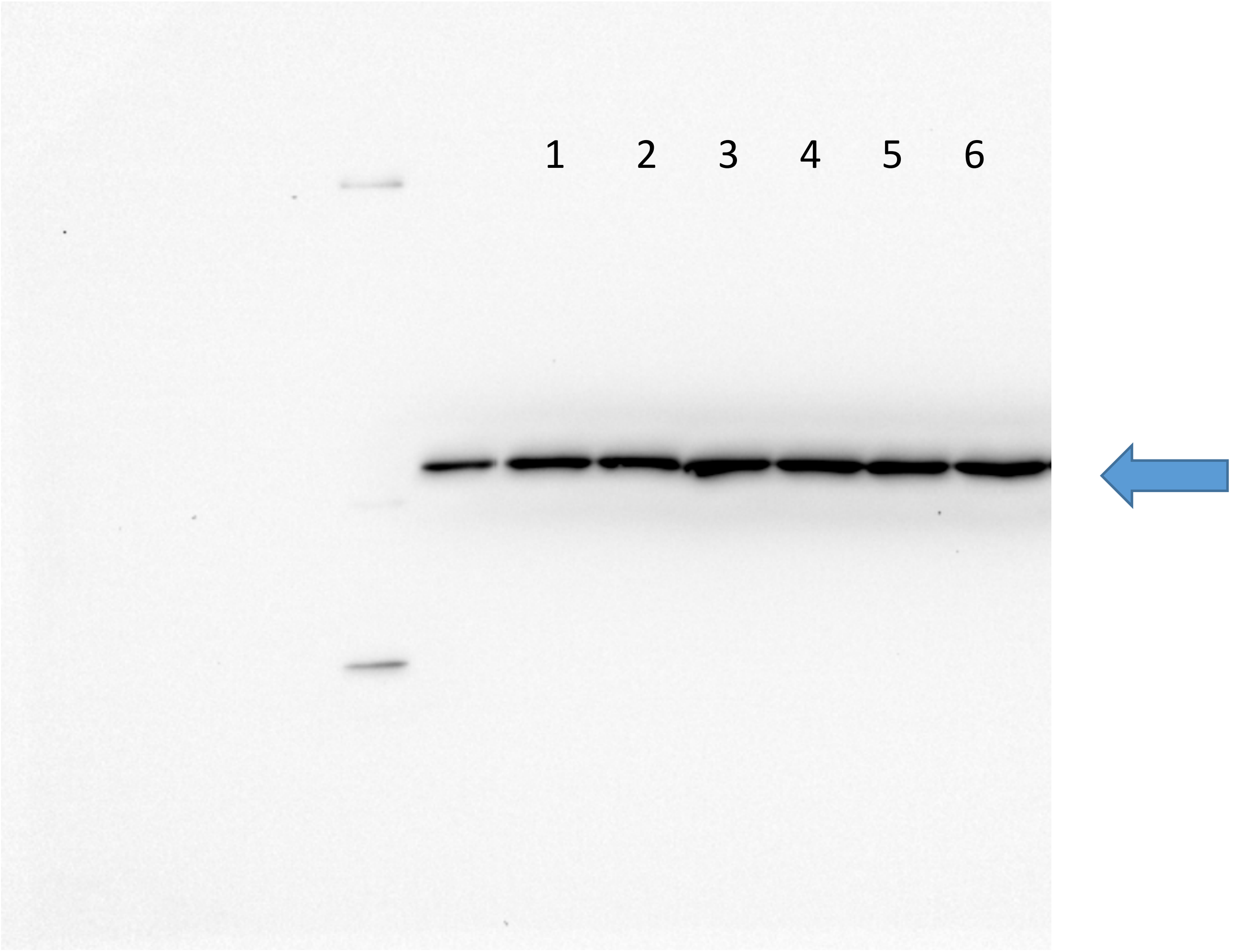

Fig3 FADS2

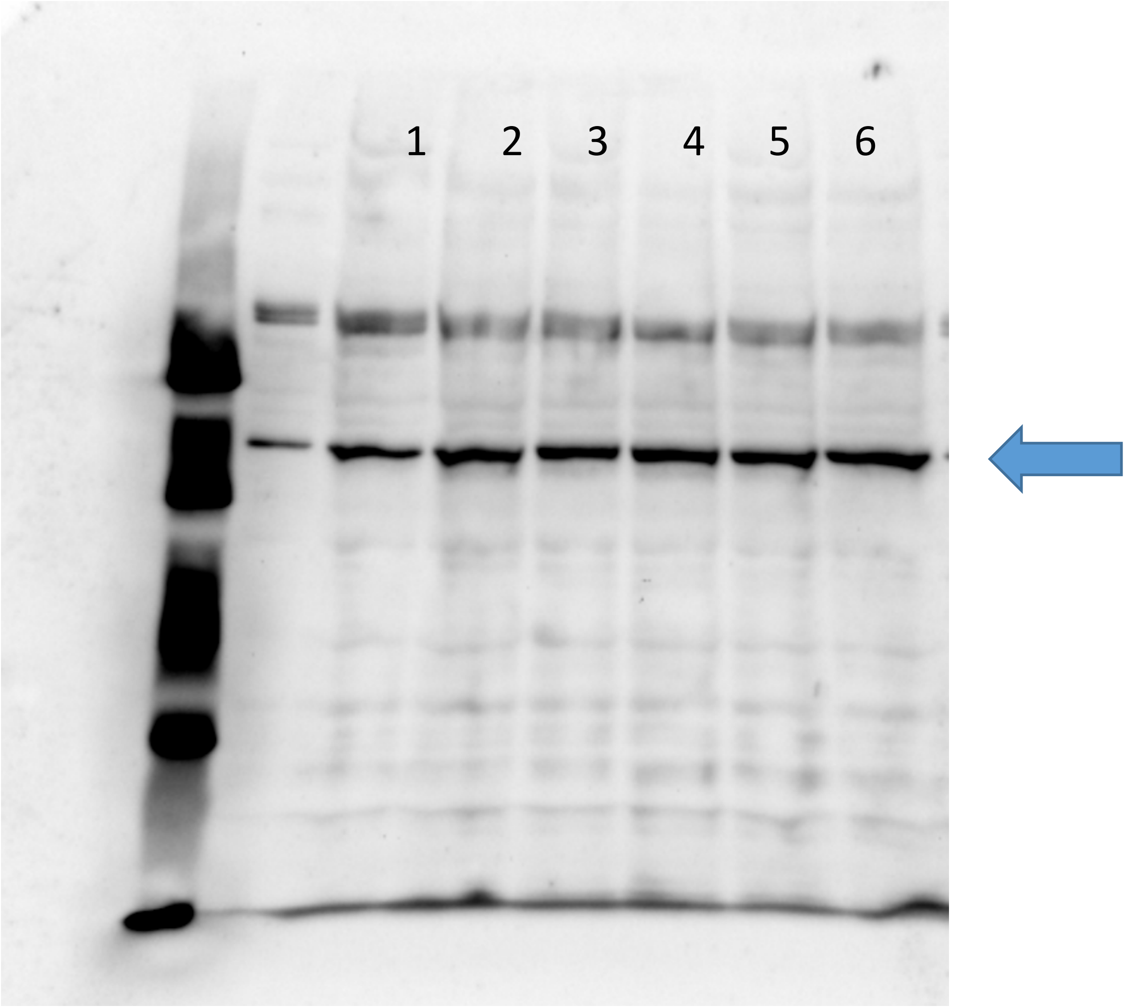

Fig3 GAPDH

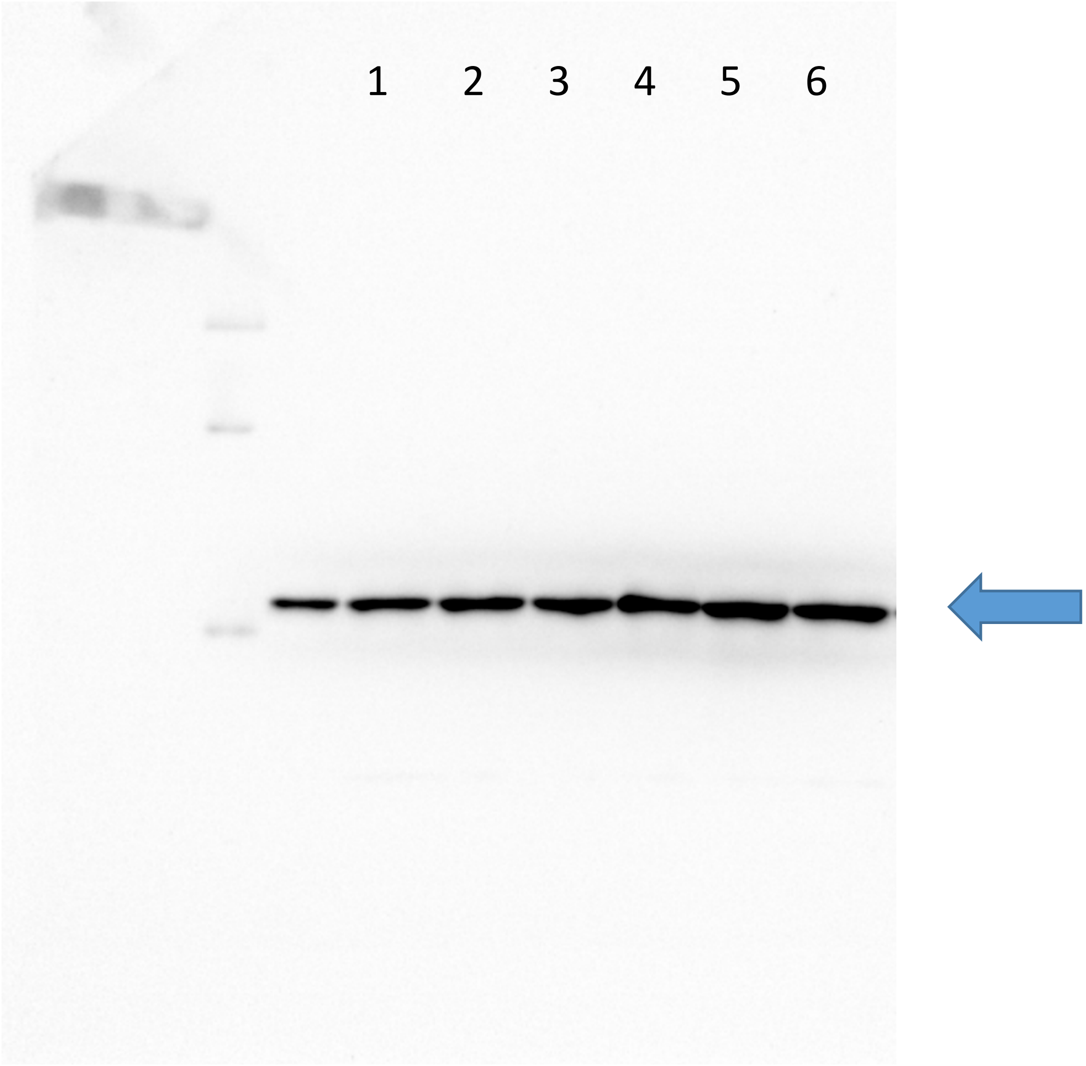

Supplement: Supplementary file 1 — Supplymentary information [file 41598_2019_46461_MOESM1_ESM.pdf]
